# Enhancement of the Fouling Resistance of Zwitterion Coated Ceramic Membranes

**DOI:** 10.3390/membranes10090210

**Published:** 2020-08-29

**Authors:** Max Storms, Abbas J. Kadhem, Shuting Xiang, Matthew Bernards, Guillermina J. Gentile, María M. Fidalgo de Cortalezzi

**Affiliations:** 1 Department of Civil and Environmental Engineering, University of Missouri, E3509 Lafferre Hall, Columbia, MO 65211, USA; miszb4@mail.missouri.edu (M.S.); ajkqmb@mail.missouri.edu (A.J.K.); 2 Department of Chemical Engineering, University of Missouri, E3509 Lafferre Hall, Columbia, MO 65211, USA; sx6cf@mail.missouri.edu; 3 Department of Chemical and Biological Engineering, University of Idaho, 875 Perimeter Drive, MS 1021, Moscow, ID 83844, USA; mbernards@uidaho.edu; 4 Department of Chemical Engineering, Instituto Tecnológico de Buenos Aires (ITBA), Av. Eduardo Madero 399, Buenos Aires 1106, Argentina; ggentile@itba.edu.ar

**Keywords:** membrane fouling, ceramic membranes, zwitterionic polymer, iron oxide

## Abstract

Ceramic membranes suffer from rapid permeability loss during filtration of organic matter due to their fouling propensity. To address this problem, iron oxide ultrafiltration membranes were coated with poly(sulfobetaine methacrylate) (polySBMA), a superhydrophilic zwitterionic polymer. The ceramic-organic hybrid membrane was characterized by scanning electron microscopy (SEM) and optical profilometry (OP). Membranes with and without polySBMA coating were subjected to fouling with bovine serum albumin solution. Hydraulic cleaning was significantly more effective for the coated membrane than for the non-coated one, as 56%, 66%, and 100% of the fouling was removed for the first, second, and third filtration cycle, respectively. Therefore, we can highlight the improved cleaning due to an increased fouling reversibility. Although some loss of polymer during operation was detected, it did not affect the improved behavior of the tested membranes.

## 1. Introduction

As global demands for drinking water increase, traditional water sources become depleted or polluted. Therefore, it is necessary to search for sustainable and economic methods, technologies, and materials for water treatment [1,2,3]. This is of particular interest for large urban populations and for small rural communities, as well as mobile applications [4,5].

Membrane filtration is a fast growing technology for water treatment thanks to the development of new membrane materials and improved fabrication over the last decades [6,7,8,9]. The need for innovative potabilization methods in a sustainable, cost-effective, and energy efficient manner can be potentially achieved with membrane technologies [10]. These technologies encompass a diverse group of processes (i.e., microfiltration, ultrafiltration, nanofiltration, and reverse osmosis) with different capabilities, and allow the removal of a wide variety of contaminants, from ions and dissolved macromolecules, to suspended colloids [11]. Thus, these processes have the potential to replace more aggressive physiochemical treatments, such as coagulation/flocculation, traditional granular filtration, and chlorine disinfection [12]. Hence, they would reduce the physical footprint of water treatment facilities, as well as streamline the water treatment process.

When a water stream is filtered, the undesirable compounds tend to accumulate near the membrane and a layer of accumulated pollutants develops on the surface, which causes permeate flux decline, diminished rejection, and higher pressure drop, increasing the operation costs of the process. Besides, the cleaning frequency becomes shorter, with the increased chance of membrane damage. In conclusion, membrane fouling must be controlled, since it has been recognized as the main limiting effect on membrane performance and therefore, the widespread application of this technology in a cost-effective and efficient way [13,14,15,16].

Fouling is considered reversible if it can be hydraulically removed (e.g., by backwashing or by turbulence) or irreversible when the foulants are physically or chemically adsorbed to the membrane surface, resulting in a permanent damage to the material that requires a more aggressive, usually chemical cleaning method to revert the permeability loss [17,18]. The origin of the fouling during natural water filtration can be related to microorganisms, organic compounds, and inorganic substances or minerals, each kind demanding a different strategy for their removal [19,20,21]. Ceramic membranes are inherently stronger and more resistant materials than polymeric, and thus more aggressive and effective chemical cleaning can be applied [22]. However, ceramics can be particularly prone to fouling by natural organic matter [23], which partially offsets the mentioned benefits. Proteins were identified as the most damaging fraction of the organic matter content, due to their high potential for irreversible adsorbing to metal oxide materials when present in a dominant concentration, as well as their capacity to magnify fouling effects due to synergistic interactions with humic substances and polysaccharides [24].

The incorporation of iron oxides into ceramic and polymeric membranes was previously performed [22,25,26,27]. This modification conveyed a set of desirable characteristics to the resulting membrane. Among these, we can mention high physical rejection, relatively low toxicity, catalytic activity, heavy metal adsorption, and potential use for disinfection [22,28,29,30,31,32,33,34].

To reduce fouling, surface modifications including zwitterion molecules to polymeric membranes have been proposed [35,36,37,38,39,40]. In particular, poly(sulfobetaine methacrylate) (polySBMA) is a zwitterion polymer that is formed by a methacrylate main chain and a sulfobetaine analogue as the pendant group, holding both positive and negative functional groups [41,42]. Since polySBMA has superhydrophilic properties [43], it was investigated as an ultralow fouling material for a variety of applications, including wound dressings [44], surface modification of polymeric membranes [45,46,47], and as an electrospun membrane material [48]. In these cases, polySBMA surface materials proved to be resistant to fouling and specifically to protein adsorption. However, there are no previous reports of surface modifications of ceramic membranes, most likely due to the inability to apply the grafting techniques used in polymers to metal oxides. Thus, the complexities of the chemical modification has thwarted the development of antifouling ceramic surfaces.

In this work, polySBMA was investigated as a coating material with the objective of minimizing fouling of an iron oxide ultrafiltration ceramic membrane, by the modification of both the mechanism and degree of fouling. The application of the antifouling layer was done by a simple surface coating; the polySBMA molecules adsorbed to the surface were expected to hinder the interaction of organic compounds in the feed solution with the membrane surface and prevent fouling in surface pore opening and pore channels, a major concern in the case of rough surfaces. The straightforward coating process can even be applied to the membrane modules in operation, if additional fouling protection would be needed.

The membrane was fabricated by the deposition of carboxylated iron hydroxide (ferroxane) nanoparticles onto alumina supports, and converted to hematite upon sintering. The hematite filter used in this work presents some advantages over previously studied systems: Low fabrication cost, the ferroxane-precursor particles are synthesized in aqueous media at moderate temperature, it is compact due to its high specific active area, operation is easy, and no sludge is produced [5,17,34]. The attachment of polySBMA to the membrane surface was performed through a simple brush coating method. Bovine serum albumin (BSA) was selected as a model foulant, representing high-molecular-weight proteins that may be found in surface waters and in biological reactors. Filtration experiments were conducted and membranes with the zwitterionic polymer coating demonstrated improved performance during three filtration stages, with increased flux recovery after cleaning to eliminate reversible fouling in comparison with the uncoated ceramic membrane. The loss of polymer during operation was also assessed.

## 2. Materials and Methods

### 2.1. Fabrication of Ferroxane Derived Ceramic Membranes

A two-step process was used to synthesize ferroxane nanoparticles. First, industrial grade FeCl_2_ (28–32% *w*/*w*), obtained from PPE Argentina S.A., was oxidized to lepidocrocite (γ–FeOOH) [41], at pH 6.8, using 3 M NaOH (Anedra, Bahia Blanca, Argentina) to avoid the acidification of the medium. Then, it was reacted with anhydrous acetic acid (Sigma Aldrich, St. Louis, MO, USA) at 70 °C to obtain ferroxane nanoparticles: Smaller iron oxide particles with a lepidocrocite-like core and an organic coated surface. The reaction with acetic acid results in a significant decrease in size of the original lepidocrocite particles due to the attack of the hydrogen bonds in their structure [47]. For the preparation of the ceramic membranes, 100 mL of a 0.15 g/L suspension of ferroxane nanoparticles were filtered through alumina filters acting as support material (1 µm nominal pore size, 47 mm diameter, 3 mm thickness, Refracton, Newark, NJ, USA) using a vacuum filtration cell (Fisher, Pittsburgh, PA, USA). Particles retained on the support formed a thin and uniform coating layer. The thickness of the iron oxide coating, based on previous studies that followed the same fabrication methodology, is approximately 15 µm [17].

The coated supports were dried at room temperature and sintered at a maximum temperature of 410 °C to produce full conversion of the ferroxane nanoparticles to hematite. The sintering process was carried out using a high temperature furnace (Vulcan 3-550, Neytech, Blommfield, CT, USA). The temperature was increased gradually at a rate of 1 °C/min, including dwelling times of 2 h at 130 °C, 3 h at 280 °C, and 4 h at 410 °C, in order to avoid cracks in the iron oxide layer due to thermal stress.

Unsupported ferroxane derived ceramics were also prepared. A concentrated suspension of the precursor particles was dried, at room temperature, and then sintered, following the same procedure as for the coated supports.

### 2.2. Modification of Ferroxane Derived Membranes Surfaces with PolySBMA

PolySBMA was synthesized according to previously described methods [46,48]. Briefly, 0.05 moles of sulfobetaine methacrylate (SBMA; Monomer-Polymer and Dajac Labs, Trevose, PA, USA) were dissolved in 100 mL of ultrapure water (resistivity 18 MΩ.cm) containing 5 mM potassium persulfate (> 99%, Acros, Geel, Belgium), as initiator, and 0.5 M KCl (Fisher, Pittsburgh, PA, USA), to control the molecular weight of polySBMA. The mixture was reacted for 5 h under nitrogen protection at 60 °C, following a previous publication [49]. PolySBMA with a molecular weight of 422 kDa was obtained and used in this work. The molecular weight of the polymer was determined by molecular sieve chromatography using a Waters 2690 Alliance high-performance liquid chromatography (HPLC) system (Milford, MA, USA) equipped with a Refractive Index (RI) detector as described elsewhere [48].

Coating of the membrane surface with polySBMA was performed using a stiff brush (Fisher, Pittsburgh, PA, USA). A thin layer of polymer, in an ultrapure water solution whose pH was adjusted to 7, was applied and spread over the membrane surface until no exposed ceramic membrane was visible to the naked eye. Then, the membranes were dried at room temperature for 24 h. Some samples received a second coating layer, repeating the previous procedure, after the first layer was dry.

### 2.3. Membrane Characterization

The pore size of the final ceramic membrane is related to the size of the precursor particles. Therefore, the particle size distribution of the ferroxane nanoparticles was measured by dynamic light scattering (DLS) using a Malvern Zetasizer Nano ZS (Malvern Instruments, Malvern, UK). The measurements were conducted on particle suspensions in ultrapure water, at circumneutral pH. Particle suspensions were diluted in order to minimize interparticle interactions during the measurements; the lowest concentration that provided acceptable signal to noise ratio, as determined by the instrument software, was used.

The specific surface area of the sintered iron oxide ceramic was measured by Brunauer–Emmet–Teller (BET) N_2_ adsorption method and the pore size calculated using the Barret, Joyner, and Halenda (BJH) model, in a SA 3100 (Beckman Coulter, Brea, CA, USA) analyzer at 77 K. Unsupported, sintered ceramic samples were fabricated for this test. Samples were degassed at 300 °C for 1 h, before the nitrogen adsorption experiment.

To assess similarities and differences, both uncoated and coated membrane surfaces were investigated by Scanning Electron Microscopy (SEM) using a Quanta FEG (FEI, Hillsboro, OR, USA). A thin layer of platinum was applied via sputter coating (K575x Sputter Coater—Emitech, Montigny-Le-Bretonneux, France) to provide a conductive surface. To extract topographical data from the surface and quantify the roughness, optical profilometry scans were obtained in a Wyko NT9100 (Veeco, Plainview, NY, USA) Vertical Scanning Interferometer (VSI). The scans were conducted on a sample area of approximately 150 µm × 100 µm. Average roughness (R_A_) and root-mean-square roughness (R_RMS_) were calculated as shown in Equations (1) and (2), respectively.

(1) $$R_{A}=\frac{1}{n}\overset{n}{\underset{i=1}{\sum }}\left|Z_{i}-\dot{Z|}\right|$$

(2) $$R_{\mathrm{RMS}}=\sqrt{\frac{1}{n}\overset{n}{\underset{i=1}{\sum }}{\left(|Z_{i}-\dot{Z|}\right)}^{2}}$$

where n is the number of measurements, Zi is the measured height (µm), and Ż is the mean height of the profile peak (µm). R_A_ and R_RMS_ (µm) were calculated from measured microscopic peaks and valleys present on the membrane surface. Both parameters employ the same individual measurements, and although R_A_ is the most commonly used, and therefore useful for comparison with other materials, R_RMS_ offers more sensibility to large deviations from the mean value [50].

### 2.4. Filtration Experiments

The permeate flux through the ceramic membranes, with and without polySBMA coating, and through the fouled membranes was determined by clean water filtration experiments in dead-end mode, in a 350 mL ultrafiltration stirred cell (Amicon Stirred Millipore, Bedford, MA, USA). The transmembrane pressure was kept constant at 103,421 Pa (15 psi) by a compressed air cylinder connected to the filtration cell.

**Figure 1:**
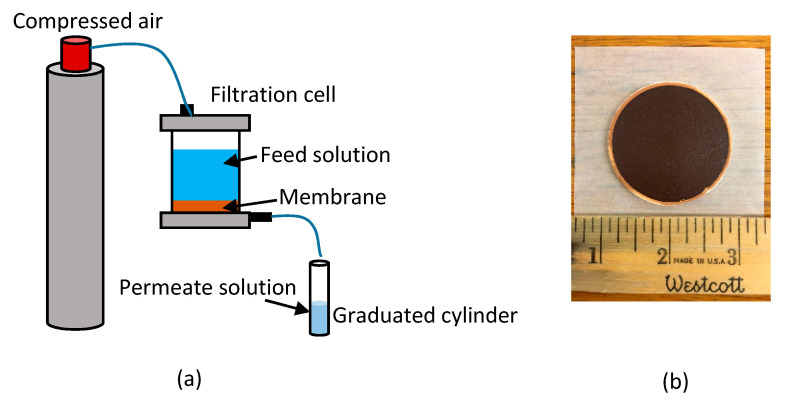
(**a**) Experimental set up for the filtration experiments. (**b**) Uncoated ceramic membrane used in the experiment.

Figure 1 shows the experimental set up used for the filtration experiments and the ferroxane derived membrane.

The permeate volume was recorded over time and the flux was calculated from the slope of the linear fit of the experimental data to Darcy’s equation:

(3) $$J=\frac{\mathrm{dV}}{\mathrm{dt}.A}=\frac{\Delta P}{\mu .{\;R}_{T}}$$

where J is the permeate flux (m^3^ m^−2^ s^−1^), A is the effective filtration area (m), ∆P is the transmembrane filtration pressure (Pa), µ is the solution viscosity (Pa.s), and R_T_ is the total membrane hydraulic resistance (m^−1^), resulting from the sum of resistances in series given by the support, the iron oxide layer, the polymer layer, and the fouling layer if present. Experimental runs were conducted in 250 mL increments: A volume of 300 mL was placed into the ultrafiltration cell, filtration was run until 250 mL of permeate was collected, to avoid data artifacts due to extremely high concentration of the feed, contaminant precipitation, and surface drying; feed solution was replenished as needed.

The relative propensity for fouling of uncoated and double-coated membranes was investigated, alongside with flux decline, hydraulic cleaning, and fouling reversibility. The experiments were carried out using bovine serum albumin (BSA) (Fischer, Pittsburgh, PA, USA), as a model of a highly fouling compound. BSA concentration in the feed was 1000 ppm, based on previous reports in the literature and on the expected fouling potential [17,40,51], in ultrapure water at circumneutral pH. Prior to the filtration tests, membranes were washed with 300 mL of ultrapure water. The fouling experiments were conducted as follows: 150 mL of BSA solution were filtered; then, the membrane was tangentially rinsed with 100 mL of ultrapure water, to simulate hydraulic cleaning as well as to determine the degree of irreversible fouling. This procedure was repeated twice to simulate the life of a membrane in operation undergoing multiple cleanings [24].

The fouling was characterized by the flux decline after each cycle of filtration, %J_f,i_, and the flux recovered after each washing, %J_w,i_, which were calculated using the following expressions:

(4) $${\%J}_{f,i}=\frac{J_{i,t=0}-J_{i,t=\mathrm{final}}}{J_{i,t=0}}$$

(5) $${\%J}_{w,i}=\frac{J_{i+1,t=0}-J_{i,t=0}}{J_{i,t=0}}$$

where %J_f,i_ is the percent flux decline after the filtration cycle “i” was completed, J_i,t=0_ is the permeate flux through the membrane at the beginning of cycle “i”, J_i,t=final_ is the permeate flux through the membrane when cycle “i” was stopped, %J_w,i_ is the percent recovery of flux for cycle “i” after hydraulic washing with respect to the flux obtained at the beginning of cycle “i”, and J_i+1,t=0_ is the permeate flux through the membrane at the beginning of the next cycle, “i+1”.

The flux decline obtained after each cycle could be reversible or irreversible, which were calculated as follows:

(6) $$\%\mathrm{rev}=\frac{J_{i+1,t=0}-J_{i,t=\mathrm{final}}}{J_{i,t=0}-J_{i,t=\mathrm{final}}}$$

(7) $$\%\mathrm{irrev}=\frac{J_{i,t=0}-J_{i+1,t=0}}{J_{i,t=0}-J_{i,t=\mathrm{final}}}$$

where %rev is the percent reversible flux decline and %irrev is the percent irreversible flux decline.

Furthermore, the loss of polySBMA during operation was determined by clean water filtration (dead-end condition) through a membrane that had received two polySBMA coatings. Permeate samples were taken at 20 mL intervals and their total organic carbon (TOC) concentration was measured using a Shimadzu TOC Analyzer, TOC-VCPH, equipped with an ASI-V auto sampler (Kyoto, Japan). The experiment was conducted in triplicates, and the total organic carbon analysis of the filtrate samples measured at least three times, so the analytical error was below 2%.

### 2.5. Analysis of Fouling Mechanism

Different models have been proposed to explain the flow reduction over time during membrane filtration. These models describe four different blocking mechanisms: Complete blocking, standard blocking, intermediate blocking, and cake filtration [51,52].

The acting mechanism depends on the relative size of the particles to the membrane pores. When particles are larger than the membrane pores, they obstruct them leading to complete blocking. On the contrary, when the particles are smaller than the average pore size, they may initially attach to their internal surface, diminishing the pore volume and giving rise to standard blocking; intermediate blocking will follow, since new particles will adsorb to previously deposited particles or to the free area that remains on the membrane surface. The last mechanism is cake filtration, which occurs when the membrane is already covered with a layer of particles that can further adhere new incoming ones.

The following expressions relate flux reduction to time for complete blocking (Equation (8)), standard blocking (Equation (9)), intermediate blocking (Equation (10)), and cake filtration (Equation (11)):

(8) $$\frac{J}{J_{0}}=e^{-\mathrm{At}}$$

(9) $$\frac{J}{J_{0}}=\frac{1}{{\left(1+\mathrm{Bt}\right)}^{2}}$$

(10) $$\frac{J}{J_{0}}=\frac{1}{1+\mathrm{At}}$$

(11) $$\frac{J}{J_{0}}=\frac{1}{\sqrt{1+\mathrm{Ct}}}$$

where J is the flux and J_0_ is the initial flux (mL/s), t is time (s), A, B, and C represent the portion of membrane blocked by deposited particles, the decrease in cross-sectional area of the pores due to adsorbed particles within them, and the influence of the formed cake that hinders the flow to pass through the membrane, respectively (s^−1^). They are expressed as:

(12) $$A=K_{A}\cdot u_{0}$$

(13) $$B=K_{B}\cdot u_{0}$$

(14) $$C=2\cdot R_{r}\cdot K_{c}\cdot u_{0}$$

where K_A_ is the blocked membrane surface per unit of total volume permeated, K_B_ is the decrease in cross-section area of the pores, due to the particles deposited on the walls, per unit of total volume permeated, K_c_^−1^ is the total volume permeated per unit of membrane area, u_0_ is the mean initial velocity of the filtrate, and R_r_ is the ratio of the resistance of the cake to the clean membrane resistance [17].

In order to elucidate the underlying mechanisms, the experimental data were fitted to the equations mentioned above using A, B, and C as adjusting parameters. The four mechanisms were tested and those that best fitted the experimental results were identified as most representative of the fouling process under the operating conditions.

## 3. Results

### 3.1. Membrane Characterization

The average hydrodynamic diameter of ferroxane nanoparticles, obtained by DLS, was 69.9 ± 17.2 nm. Nitrogen adsorption isotherms of unsupported iron oxide ceramics showed a BET specific surface area of 72.47 ± 2.01 m^2^/g. This relatively high specific surface area suggests a rough, tortuous pore structure for the iron oxide layer. The average pore size, calculated using the BJH model, was 40 ± 19 nm, in good agreement with previously reported values [17], placing the asymmetric membrane in the ultrafiltration range [11].

**Figure 2:**
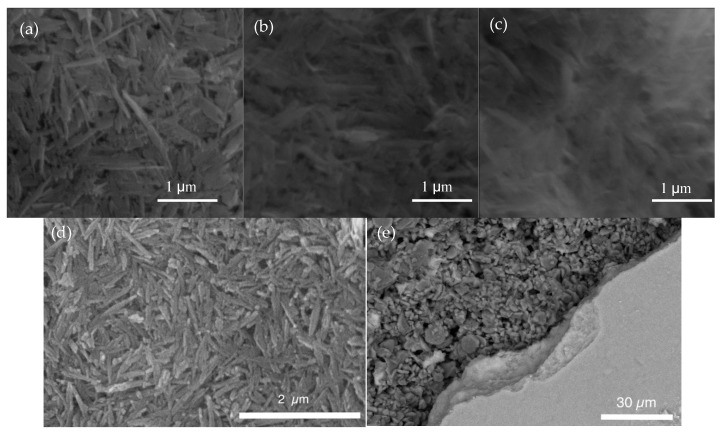
SEM images of: (**a**) Membrane with no polySBMA coating, (**b**) membrane with one polySBMA coating layer, (**c**) membrane with two polySBMA coating layers, (**d**) top view of iron oxide layer with polymer coating, after use, (**e**) iron oxide layer partially removed, exposing support material.

SEM images of the top surface of the membranes with and without polySBMA coatings are shown in Figure 2. In Figure 2a, the characteristic elongated shape of the ferroxane particles is still visible providing the membrane with a highly rough appearance [34]. Figure 2b,c show the progressive masking of the surface features with an increasing number of polySBMA layers, implying that the coating had a smoothing effect on the membrane surface. Figure 2d shows the membrane after use, and evidences the loss of excess polymer during use, as most of the original features are visible. A section of the iron oxide coating layer was scratched off the surface, exposing the underlying support material which can be observed in Figure 2e. Based on previous work by the group following a similar fabrication protocol, the thickness of the iron oxide layer was estimated to be approximately 15 µm; the SEM image suggests a slighter thicker effective filtration layer, related to an increase in the concentration of ferroxane particles deposited by filtration [17].

**Figure 3:**
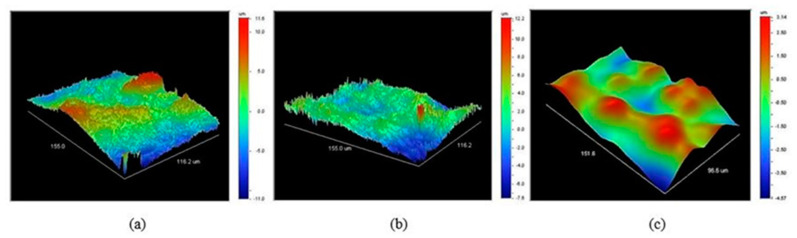
Optical profilometry 3D renders of: (**a**) Clean membrane surface, (**b**) membrane surface with one polymer coating, (**c**) membrane surface with two polymer coatings.

The optical profilometry scans of the membrane surfaces with and without polySBMA coating are shown in Figure 3. In agreement with the SEM images, the smoothing effect is evident and can be quantified by this technique. Comparison of Figure 3a,b suggests that the first coating treatment only produced a modest smoothing of the surface. Figure 3c corresponds to a membrane that underwent two coating treatments and the consequent difference in the morphology is more marked; the elongated, needle-like ferroxane nanoparticles disappeared and were replaced by a more uniform surface.

**Figure 4:**
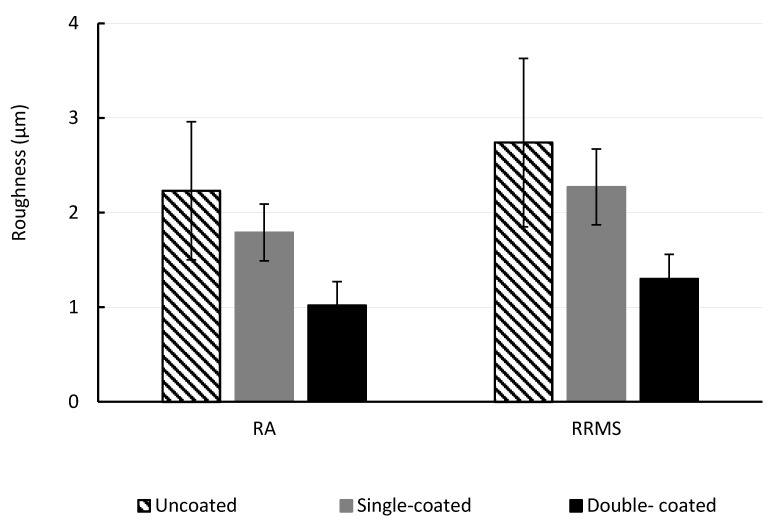
Data comparison of roughness values collected using optical profilometry of uncoated, single-coated, and double-coated membrane surfaces (R_A_: Average roughness, R_RMS_: Root-mean-square roughness).

Surface roughness is important as it affects the fouling potential of the membrane. Foulants can accumulate in the valleys created by pore entrances that also shield them from removal during hydraulic cleaning. The surface roughness was determined by average (R_A_) and root mean square (R_RMS_) roughness parameters, in order to quantify the changes in topography and the results are shown in Figure 4. Five different areas of each membrane sample were investigated. For membranes without polySBMA coating, R_A_ was 2.23 ± 0.73 µm and R_RMS_ was 2.74 ± 0.89 µm. For membranes with a single polySBMA coating, R_A_ was 1.79 ± 0.3 µm and R_RMS_ was 2.27 ± 0.4 µm. For membranes with two polySBMA coatings, R_A_ was 1.02 ± 0.25 µm and R_RMS_ was 1.30 ± 0.26 µm.

The changes in surface morphology observed from a membrane with no polymer coating to a membrane with a single coating corresponds to a reduction of 19.7% in R_A_ and 17.2% in R_RMS_. The second coating layer produced a further decrease to 54.2% in R_A_ and 52.5% in R_RMS_.

### 3.2. Filtration Experiments

Clean water flux was measured for uncoated and double-coated membranes at a transmembrane pressure of 103,421 Pa. Average measured fluxes were 222.0 L/(m^2^·h) for membranes without coating and 164.7 L/(m^2^·h) for membranes with a double-polySBMA coating, which accounts for a reduction of 26%. As expected, a decrease in the permeability of the membrane with polySBMA coating was observed, though an acceptable flux was still achieved.

Using Equation (3), we could obtain the resistance to flux to be 1.88 × 10^12^ m^−1^ for the uncoated membrane and 2.54 × 10^12^ m^−1^ for the membrane with two coatings. Thus, the polymer increased the resistance in 6.65 × 10^11^ m^−1^.

### 3.3. Membrane Fouling

**Figure 5:**
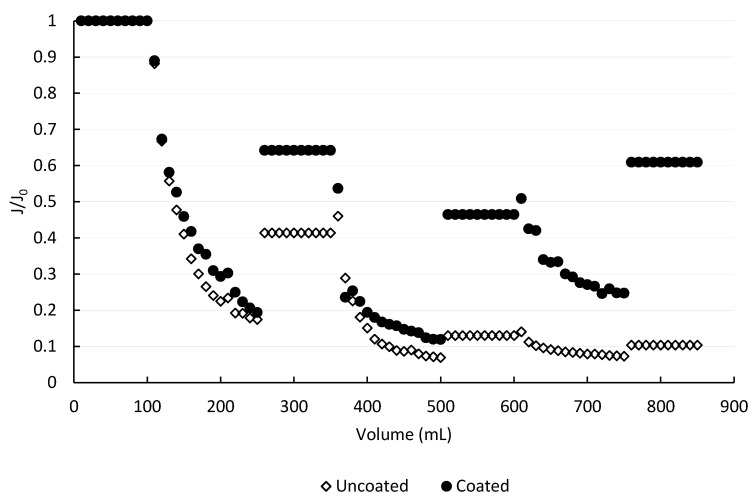
Comparison of the fouling behavior between two membrane samples, with and without polymer coating, in ultrapure water, neutral pH, ΔP = 103,421 Pa.

An assessment of the fouling was obtained by comparing the calculated permeability for the clean membrane and for the fouled membrane, studying flux decline, hydraulic cleaning, and fouling reversibility. Figure 5 shows the change in flux for an uncoated and a double-coated membrane during BSA filtration due to fouling, in ultrapure water at neutral pH. In both cases, three filtration-fouling cycles were performed, with tangential washing between them. The degree of fouling reversibility was obtained by comparison of the recovered flux to the initial flux for each cycle. Three or more runs always with different membranes were performed.

Filtration of the first 250 mL of the BSA solution through the uncoated membrane resulted in a flux decrease to 0.174 of the initial flux. After hydraulic cleaning, a recovery to 0.41 of the initial flux was obtained. Each washing step was followed by filtration of 100 mL of clean water in order to assess the permeability of the fouled membrane. It is important to note that during clean water filtration, permeate flux remained constant for all experiments, indicating that the residual foulant layer was stable and did not undergo any further changes, such as compaction or resuspension. In a second cycle, only 150 mL of BSA solution were filtered through the membrane before the flux reached a minimal level of 0.07 J_0_, at which operation was not practical due to the extremely low permeate flow. Water rinsing of the membrane surface provided modest relief, with flux increasing only to 0.13 J_0_ after treatment. The third cycle resulted in flux to decline to a similar value after filtration of 150 mL of BSA solution; hydraulic cleaning showed almost negligible improvement. The fraction of the fouling that was reversed by the hydraulic washing also dropped from the first to the second cycle, from 29% to 18%, as the fouling layer became more stable and strongly bound to the surface. Although the percentage of recovery in the third cleaning cycle appears to improve, it is an artifact of the low permeability values and the overall negligible changes.

When a polySBMA coated membrane was subjected to the same cycle of filtration and cleaning, important differences were observed. The interaction between the clean membrane and the BSA solution led to a similar decrease in flux after the first 250 mL were filtered, to about 0.20 J_0_. However, the recovery due to hydraulic cleaning was much higher than for the uncoated material, reaching 0.64 J_0_. The second and third cycle of filtration, both consisting of 150 mL of feed solution, resulted in flux declines to 0.12 J_0_ and 0.25 J_0_, respectively. This was expected given the high concentration of BSA in the feed solution and the dead-end configuration of the experimental set-up. However, hydraulic cleaning was much more effective for the coated membrane. The reversible fraction of the fouling was 56%, 66%, and 100%, for the first, second and third treatment cycle, respectively. Unlike the results with the uncoated membrane, an upward trend of reversibility was identified. The experimental observation was consistent with the occurrence of some degree of BSA irreversible adsorption to the membrane surface during the first cycle of operation, but signaled that additional retained molecules were loosely bound and therefore were susceptible to removal by physical methods, i.e., hydraulic cleaning. In fact, the clean water flux after the third wash reached levels around 10% above the one observed after the second filtration cycle. This result showed the inherent randomness in the formation of the foulant layer, since the third cycle of filtration appeared to produce a more unstable foulant layer than during the second cycle. Another source of variation may be the cleaning procedure, when the membrane was removed from the cell and cleaned with ultrapure water laboratory squeeze bottle to simulate the tangential flow, which could lead to unintentional variations in intensity of the cleaning.

The calculated fouling parameters are presented in Table 1. It can be observed that, although the flux decline in each fouling step, calculated by Equation (4), was very similar for both kind of membranes (over 80% in the primary and secondary, and over 40% in the tertiary), the recovery, calculated by Equation (5), was always much higher when the membrane was coated. This fact can be correlated with the higher amount of reversible fouling linked to the smoother surface.

### 3.4. Determination of Fouling Mechanism

**Figure 6:**
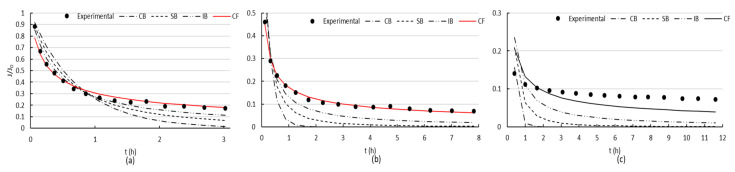
Uncoated membrane: Experimental data for: (**a**) First, (**b**) second, and (**c**) third fouling stage. The red adjusting curves indicate cake filtration as acting mechanism, with adjusting coefficient C: (**a**) (0.00269 ± 0.00016) s^−1^ and (**b**) (0.00908 ± 0.00021) s^−1^.

When filtrating BSA through the uncoated membrane, a sharp reduction in the flux was initially observed (Figure 5), which is an indication of the presence of blocking [51]. This tendency evolved into a slightly flattening of the slope, characteristic of cake formation. After the first cleaning, flux was partially recovered, and during the second filtration cycle, a sharp reduction in the flux was followed by a steady flattening of the slope, as consequence of the rapid cake formation. The second cleaning was less effective than the first one. Afterwards, during the third filtration cycle, flux did not vary considerably since it was always low, and no active mechanism could be determined for this last stage. Figure 6 presents the fittings of the four evaluated mechanisms. The modeling showed that cake formation was the mechanism that best adjusted the experimental data for the two first stages of fouling (Figure 6a,b) and no mechanism could adjust correctly the last stage.

**Figure 7:**
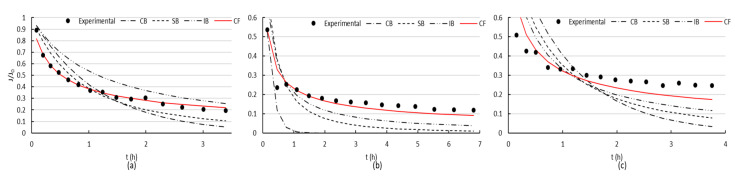
Coated membrane: Experimental data for: (**a**) First, (**b**) second, and (**c**) third fouling stage. The red curves indicate cake filtration as acting mechanism, with adjusting coefficient C: (**a**) (0.00162 ± 0.00007) s^−1^, (**b**) (0.00492 ± 0.00045) s^−1^, and (**c**) (0.00239 ± 0.00031) s^−1^.

When filtrating BSA through the membrane with two polymeric coatings, a reduction in flux was marked, but flux recovery was always higher (Figure 5). Figure 7 presents the fittings of the four evaluated mechanisms. Similar to the uncoated surface, cake formation was the mechanism that best adjusted the experimental data.

It can be clearly seen (Figure 6 and Figure 7) that both uncoated and coated membranes offered the same pattern for the first and second fouling mechanisms. The slope flattening occurred after 1.4 h approximately for the first fouling and after 1.6 h for the second. However, for the third fouling, both membranes presented different mechanism patterns. When using the coated membrane, a sharp reduction in flux occurred at the beginning, followed by a flattening of the slope after 43 min, typical of cake filtration. On the contrary, only a mild decrease in flux was observed for the uncoated membrane, which may be the effect of intense fouling present, and the models were not able to adjust the data obtained in the filtration experiments.

### 3.5. Polymer Loss

Since the polymer was simply deposited on the ceramic surface, it could be lost due to leakage through the membrane. The molecular weight of the polySBMA, 422 kDa, suggested that it should be mostly retained by the ferroxane derived membrane, whose molecular weight cut off (MWCO) was determined to be 180 kDa [34]. However, the molecule structure affects the rejection level that can be expected. Usually, dextrans, which are branched molecules, are used as test molecules in the determination, following the ASTM standard method, Publication E1393-90. Nonetheless, a linear molecule, like polySBMA, is likely to pass through the membrane even at sizes exceeding the reported MWCO.

**Figure 8:**
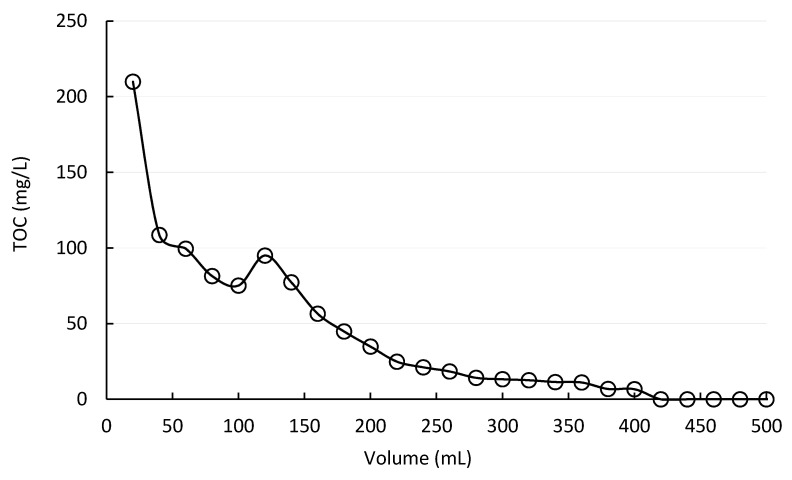
Poly(sulfobetaine methacrylate) (polySBMA) concentration in the permeate, as total organic carbon, during dead-end filtration of clean water with a newly coated membrane (ΔP = 103,421 Pa, ultrapure water, neutral pH).

The loss of polySBMA during operation was studied. Figure 8 shows a typical curve obtained in these tests.

The total polySBMA mass leaked by the membrane during clean water filtration was calculated to be 20 mg of polymer as TOC. However, an accurate assessment of the fraction of polymer loss was not feasible, due to the non-quantitative nature of the coating technique. We observed that 20% of the total loss occurred very early, in the first 20 mL of operation, and reached a non-detectable level after 400 mL of operation. Nonetheless, this fact did not seem to affect the effectiveness of the polySBMA coating, mainly related to flux recovery and fouling reversibility. All the membranes tested were washed with 300 mL of ultrapure water prior to fouling experiments. An improvement of antifouling behavior was evident during the three fouling cycles, as well as in flux recovery. Adsorption of the polymer molecules to the iron oxide surface was stronger than polymer-polymer interaction forces, and while the excess material was washed away in the initial stages of the filtration, a thin layer of polymer remained on the surface and was responsible for the antifouling properties observed.

## 4. Discussion

The ceramic membranes were fabricated in the laboratory taking special care concerning two key points; first, obtaining uniform size distribution of the ferroxane nanoparticles (69.9 ± 17.2 nm obtained by DLS), since this distribution determined the membrane pore size (40 ± 19 nm); and second, the selection of a compatible support material in order to enhance its adherence to the hematite. The method selected for covering some membranes with polySBMA had to be simple, economic, and effective without requiring any pretreatment of the membrane. Still, there is a need to improve this coating process, which proved initially to be challenging due to the high surface roughness of the material, preventing the formation of an effective layer. An optimization of the coating parameters (e.g., polymer concentration) would minimize the polymer mass initially required per unit surface of treated membrane, as well as reduce the leakage during the first stages of operation. The addition of one or two polymer coatings resulted in complete coverage of the membrane with the subsequent surface smoothing, as observed by SEM imaging and optical profilometry scanning. Besides, the membrane permeability was reduced by the polymer addition since it accumulated in the pore valleys, as indicated by the membrane surface flattening. However, this decrease was not restrictive, complete blockage did not occur and the flux was still acceptable.

The tested membranes, both with and without the polymeric coating, were prone to organic fouling. In the case of the uncoated membranes, strong attractive forces, between BSA and hematite promoted adsorption onto the surface at neutral pH [17]. After the cleanings, the flux recovery was not significant, evidencing the strong adsorption of BSA to the membrane surface, as well as the need of removing irreversible fouling. Surface charge of the iron oxide was previously measured and resulted very negative (<−20 mV) at circumneutral pH levels [17,53]. The isoelectric point of BSA was reported to be 4.7 [54,55]. Despite some degree of electrostatic repulsion was expected, fouling still prevailed due to the protein ability to develop hydrogen bonds with the iron oxide [56], resulting in irreversible fouling that could not be prevented. Two successive fouling steps developed, the first one, when attraction between the polar groups of the BSA and the hydrophilic membrane predominated and the second one, when new incoming foulant molecules were attracted to the previously deposited ones [20,57,58,59,60]. The former is mainly responsible for irreversible fouling, whereas the latter is more likely to be related to reversible fouling. Besides, the interactions among foulant molecules affect the integrity of ceramic membranes, which also occurs to polymeric membranes [20].

Flux data modeling showed that cake formation was the mechanism that best adjusted the experimental data (Figure 6) for the two first stages of fouling. This finding may surprise in a first instance due to the small size of the foulant, with a hydrodynamic diameter of only 3.48 nm [61]. However, the high concentration of BSA in the solution, the high specific surface area (72.47 ± 2.01 m^2^/g) exposed to the solution, the elongated and small pores (40 ± 19 nm), the rough and tortuous surface, together with the presence of attractive forces between the hydrophilic iron oxide and the protein with polar groups, were responsible for the fast development of a layer of foulant adsorbed to the iron oxide, that constituted a concentration polarization layer. The high degree of irreversible fouling, especially after the first and second cleaning procedures, is another proof of cake formation as the acting mechanism, combining adsorption on both surface and pore walls. In real operation systems, it is important to notice that the actual size, conformation, and stability of proteins are affected by the solution chemistry, ionic strength, pH, attraction and association with water molecules and electrolytes, and concentration. Thus, the acting mechanism, which depends on the relative size of the particles to the membrane pores, will be affected, in turn, by this variation.

The addition of sufficient polySBMA coatings resulted in less aggressive fouling and the fouling that did occur was reversible to a larger extent. The primary reason for this improvement of the fouling behavior in the coated membranes was the zwitterionic properties of polySBMA. The alternating charges of the zwitterionic polySBMA molecules allowed for enhanced interaction between polymer and water via electrostatic attraction. The high affinity for water molecules resulted in a strong hydration layer close to the membrane, preventing BSA molecules from easily interacting, covering, and attaching to its surface. This hindered attraction between BSA and the membrane surface minimized protein adsorption, increasing fouling reversibility and flux recovery. Furthermore, the surface morphology modification induced by the polymer coating, from rough to smooth, was likely a contributing factor to the antifouling behavior observed, and might enhance the membrane lifespan.

When filtrating BSA through the membrane with two coatings, the flux recovery after cleaning achieved better results than in the case of the membrane with no coating, and fouling was reversible to a higher extent (Figure 5). This clearly indicated less attractive forces between the membrane coated with polySBMA and the BSA, which combined with a smoother surface, improved the performance of the material. The superhydrophilic zwitterionic interface on the iron oxide proved to resist the nonspecific adsorption of proteins; giving a better performance facing fouling, though smaller flux was obtained due to pore occlusion by the polymer that led to a diminished initial permeability. Cake formation was the mechanism that best adjusted the experimental data (Figure 7), due to the high concentration of BSA in the solution. In all cases, hydraulic washing should be carefully performed, since an excessive compression of the irreversible fouling layer can aggravate the loss of permeability.

The loss of polySBMA during operation was a possibility due to two main reasons. First, the polymer was not covalently bonded, but rather deposited on the surface of the membrane. Second, the uncoated membrane was rough and the polymer had high viscosity, making it necessary to use an excess of polymer, following no stoichiometric relationship. Besides, this loss was of high concern, due to the potential contamination of the produced permeate. During operation, although some polymer loss was detected, the modification greatly improved the type of prevailing fouling as well as the flux recovery. This evidence can be because adsorption of the polymer molecules to the iron oxide surface is stronger than polymer-polymer interaction forces, and while excess material is easily removed, a thin and uniform monolayer remained on the surface and was responsible for the antifouling properties observed.

## 5. Conclusions

Coating of ceramic membranes derived from ferroxane nanoparticles with polySBMA helped not only to improve membrane antifouling performance after a simple tangential washing, but also to achieve a higher flux recovery. The coated membranes were fouled less aggressively, offering an optimistic view to improving fouling performance. In addition, the fouling was reversible to a higher extent. Some loss of polySBMA for the new membranes during the first stages of operation was detected; nevertheless, the membrane retained the improved antifouling properties during several cycles of filtration and hydraulic washing.

When the membrane had no coating, cake formation was the fouling mechanism due to the high concentration of protein, the high specific surface area and small pores, the rough and tortuous membrane surface, and the attractive forces developed between the iron oxide and the BSA. Analogously, the same mechanism was attributable when the polySBMA coating was present due to the high concentration of BSA in the solution.

An optimization of the coating parameters would minimize the polymer mass required per unit surface of treated membrane, as well as reduce the polymer leakage for the new membranes. Besides, due to the simplicity and effectiveness of the approach, the non-covalent coating of ceramic membranes with polySBMA is a practical strategy for extending the length of operation cycles in the filtration of highly fouling feed solutions.

This work demonstrated the beneficial effect of a simple coating procedure on reducing fouling. The coating can be applied on site, directly to an already manufactured module, with minimal investment. The potential initial loss of low amounts of polymer with the permeate may be problematic for some applications, but generally accepted for the pretreatment step of heavily polluted industrial wastewaters or raw municipal wastewater.

## Figures and Tables

**Table 1:** Flux decline due to total, reversible, and irreversible fouling for uncoated and polySBMA coated membranes.

	Uncoated Membrane				PolySBMA-Coated Membrane			
Cycle	Flux Decline after Filtration	Flux Recovered after Washing	% Reversible	% Irreversible	Flux Decline after Filtration	Flux Recovered after Washing	% Reversible	% Irreversible
1_st_	83%	41%	30	70	81%	64%	56	44
2_nd_	83%	31%	18	82	81%	72%	66	34
3_rd_	44%	80%	54	46	47%	131%	100	-
